# Virtual reality-based interventions for schizophrenia

**DOI:** 10.1192/j.eurpsy.2023.129

**Published:** 2023-07-19

**Authors:** M. Nordentoft

**Affiliations:** MentalHealth Center Copenhagen, Copenhagen University Hospital, Hellerup, Denmark

## Abstract

**Abstract: Background:**

Traditional psychotherapeutic interventions show small to moderate effect in treating psychotic symptoms. Virtual reality (VR) assisted treatments has the potential of advancing current psychotherapies for psychotic symptoms by creating virtual environments that can elicit responses (e.g., thoughts, feelings, behaviours) mirroring real-world settings. This presentation will highlight the current research initiatives using virtual reality-based interventions targeting positive and negative symptoms in patients with psychosis.

**Results:**

Main findings from the pilot-studies and randomized clinical trials on computer-based and immersive VR-interventions demonstrate preliminary evidence of VR-based psychotherapy for treating auditory hallucinations and paranoia with large effect sizes (Cohens d= 0.75-0.80). Additionally, pilot data has provided indications as to VR-psychotherapy being feasible and acceptable in treating negative symptoms and may have a large effect on participants achieving their goals and potentially in reducing negative symptoms. No adverse effect has been found related to the VR-interventions.

**Discussion:**

The promising findings on VR-based interventions for psychosis calls for large-scale randomized clinical trials consolidating the evidence for the effect in treating positive and negative symptoms in psychotic disorders. Cost-effectiveness of these short-term VR-based interventions are essential to inform scalability and implementation. Finally, most of the studies target patients in more chronic/treatment resistant phases of psychosis highlighting the need to investigating the effect in earlier phases of psychosis, that is, first-episode and potentially clinical high-risk states.

**Disclosure of Interest:**

None Declared

